# Genome-wide identification of the Tubby-Like Protein (TLPs) family in medicinal model plant *Salvia miltiorrhiza*

**DOI:** 10.7717/peerj.11403

**Published:** 2021-05-12

**Authors:** Kai Wang, Yating Cheng, Li Yi, Hailang He, Shaofeng Zhan, Peng Yang

**Affiliations:** 1 Hunan Province Key Laboratory for Antibody-based Drug and Intelligent Delivery System, School of Pharmaceutical Sciences, Hunan University of Medicine, Huaihua, China; 2 Department of Respiratory Medicine, the First Affiliated Hospital of Guangzhou University of Chinese Medicine, Guangzhou, China; 3 Hunan Provincial Key Laboratory for Synthetic Biology of Traditional Chinese Medicine, Hunan University of Medicine, Huaihua, China

**Keywords:** Tubby-Like Proteins, TLPs, Medicinal plant, *Salvia miltiorrhiza*, Abiotic stress

## Abstract

Tubby-Like Proteins (TLPs) are important transcription factors with many functions and are found in both animals and plants. In plants, TLPs are thought to be involved in the abiotic stress response. To reveal the potential function of TLPs in the medicinal model plant *Salvia miltiorrhiza*, we identified 12 *S. miltiorrhiza* TLPs (SmTLPs) and conducted a comprehensive analysis. We examined SmTLP gene structure, protein structure, phylogenetics, and expression analysis. Our results show that all SmTLPs, except SmTLP11, have a complete typical Tub domain. Promoter analysis revealed that most SmTLPs are involved in hormone and abiotic stress responses. Expression analysis revealed that the 12 SmTLPs could be divided into three categories: those specifically expressed in roots, those specifically expressed in stems, and those specifically expressed in leaves. Additional studies have shown that SmTLP10 may play an important role in the plant cold resistance, while SmTLP12 may be involved in the *S. miltiorrhiza* ABA metabolic pathway. Our study represents the first comprehensive investigation of TLPs in *S. miltiorrhiza*. These data may provide useful clues for future studies and may support the hypotheses regarding the role of TLPs in plant abiotic stress process. All in all, we may provide a reference for improving *S. miltiorrhiza* quality using genetic engineering technology.

## Introduction

Tubby-Like Proteins (TLPs) are widely distributed in living creatures and have diverse functions (Ikeda, Nishina & Naggert, 2002; Wang, Xu & Kong, 2018). Previous studies have indicated that TLPs exist in all eukaryotes, from single-celled to multicellular organisms (Wang, Xu & Kong, 2018). The TLP family shares a common tertiary structure, with a beta barrel structure packed around an alpha helix in the central pore (Yulong et al., 2016). Each TLP is characterized by a signature tubby domain at its C-terminus. The tubby domain shares 55% to 95% identity across various species and is thought to be significant for the proper folding, solubility, and subcellular localization of TLPs, as well as other important functions (Ikeda, Nishina & Naggert, 2002; Kim et al., 2017; Yang et al., 2008). In contrast to the conserved C-terminal tubby domain, motifs at N-terminus of TLPs (e.g., F-box, WD40 repeat, or SOCS box) differ greatly across species. According to previous studies, TLPs can be divided into three classes based on phylogenetic trees (Gagne et al., 2002; Lai et al., 2012). Although plants have more TLPs than do animals, the N-termini of animal TLPs are highly divergent while the majority of plant TLPs contain N-terminal F-box domains and are relatively conserved in this region (Gagne et al., 2002; Lai et al., 2012).

TLPs are thought to function as transcription factors, but the specific mechanism was unknown (Boggon et al., 2000). First identified in obese mice at the end of the last century, TLPs have been shown to play a dominant role in the maintenance and functioning of neurons during post-differentiation and TLP mutations can lead to infertility, insulin resistance, and retinal degeneration with hearing loss (Coleman & Eicher, 1990; Heckenlively et al., 1995; Kleyn et al., 1996; Noben-Trauth et al., 1996). TLPs’ roles in animals have been extensively studied, while TLP research in plants has mainly focused on the response of plants to biotic and abiotic stresses (Lai et al., 2004; Liu, 2008; Wang, Xu & Kong, 2018; Yang et al., 2008; Yulong et al., 2016; Zhang et al., 2020). In *Arabidopsis thaliana*, the TLP gene family contains 11 genes (*AtTLP1–AtTLP11*) (Lai et al., 2004). Previous research indicates that *AtTLP9* is involved in the response to salt and drought stress and that *AtTLP3* is involved in the regulation of the ABA signaling pathway during seed germination (Bao et al., 2014; Lai et al., 2004). In *Oryza sativa,* the *TLP* gene contains 14 members (*OsTLP1–OsTLP14*) and expression profiling analysis revealed that *OsTLP14* plays an important role in seed germination and seedling formation (Liu, 2008). In *Cicer arietinum*, overexpression of *CaTLP1* enhances tolerance to salt, drought, and oxidative stress (Wardhan et al., 2012). *Malus domestica* TLPs play an important role in stress response and the expression of *MdTLP7* was reported to enhance abiotic stress tolerance in Arabidopsis (Xu et al., 2019).

*Salvia miltiorrhiza* is an emerging model plant in traditional Chinese medicine research and is widely cultivated (Cheng, 2006; Xu et al., 2016c; Zhang et al., 2014). Abiotic stress has a serious impact on the cultivation of plants, so improving *S. miltiorrhiza*’s stress resistance is important. Previous studies have explored the role of *TLP* family genes in stress resistance in plants including *A. thaliana*. However, the role of TLP family genes in *S. miltiorrhiza* remains unknown. The publication of the *S. miltiorrhiza* genome provides an opportunity to perform a genome-wide analysis of the *TLP* gene family to clarify their evolutionary history and explore their functional mechanisms (Xu et al., 2016a). In this study, we identified and analyzed 12 *SmTLP* genes in *S. miltiorrhiza*. We analyzed their gene and protein structures and predicted their *cis*-activating elements. Using qPCR, we analyzed the role of *SmTLPs* in plant abiotic stress. These results lay the foundation for further elucidation of the roles of the *SmTLPs* family in stress resistance.

## Material and Methods

### Data collection

Initially, based on the keyword Tubby like protein, already-known TLP protein sequences from *A. thaliana, S. lycopersicum*, and *O. sativa* were retrieved from the NCBI database (https://www.ncbi.nlm.nih.gov/) (Table S1). The *S. miltiorrhiza* genome (published) was downloaded from the China National Traditional Chinese Medicine Data Center (ftp://danshen.ndctcm.org:10402/) (Xu et al., 2016a).

### Identification of TLPs in *S. miltiorrhiza* data collection

We used two methods to accurately identify TLPs in *S. miltiorrhiza*. First, we used the online software iTAK (http://itak.feilab.net/cgi-bin/itak/index.cgi) to directly identify 12 candidate *SmTLPs* (Zheng et al., 2016). Then, based on the hidden Markov model (HMM) of the Tubby domain (PF01167) obtained from the Pfam database (http://pfam.xfam.org/), we also identified 12 candidate *SmTLPs* using Hmmsearch (3.2.1) (Potter et al., 2018). Next, we used the Web CD-Search Tool (https://www.ncbi.nlm.nih.gov/Structure/bwrpsb/bwrpsb.cgi) to identify and confirm the domains of candidate *SmTLPs* identified by the two methods. Finally, we identified 12 *SmTLPs* and named them *SmTLP1–SmTLP12*. The *SmTLPs*’ physicochemical characteristics were analyzed using the ProtParam tool (http://web.expasy.org/protparam/). Characteristics included the grand average of hydropathicity (GRAVY), instability index, theoretical isoelectric point (*p* I), and molecular weight (Mw). The WoLF PSORT (https://wolfpsort.hgc.jp/), Busca (http://busca.biocomp.unibo.it/) and Plant-mPLoc (http://www.csbio.sjtu.edu.cn/bioinf/plant-multi/) were used to predict the subcellular localization of the SmTLPs in *S. miltiorrhiza*.

### Analysis of gene structure and protein structure

TBtools was used to analyze the gene structure of *SmTLPs* (Chen et al., 2020). Conserved motifs in *SmTLPs* were identified using the MEME tool (http://meme-suite.org/tools/meme) and the following criteria: maximum number of motifs were set to 10; the minimum motif width was 6; and the maximum motif width was 200. Other parameters were set to default. Tubby domain protein alignment was performed using Clustal Omega (https://www.ebi.ac.uk/Tools/msa/clustalo/) and verified by MEGA X (Kumar et al., 2018; Sievers & Higgins, 2018; Sievers et al., 2011). The SWISS-MODEL (https://www.swissmodel.expasy.org/) was used to construct the *SmTLPs* homologous protein model using the default parameters (Bienert et al., 2017; Guex, Peitsch & Schwede, 2009; Waterhouse et al., 2018).

### Multiple sequence alignment and phylogenetic tree construction

All *S. miltiorrhiza, A. thaliana*, *S. lycopersicum*, and *O. sativa* TLPs were pooled into the Clustal Omega tool (https://www.ebi.ac.uk/Tools/msa/clustalo/) to perform multiple sequence alignments (Sievers & Higgins, 2018; Sievers et al., 2011). MEGA X (version 10.0.5) was used to perform a phylogenetic analysis of the aligned protein sequences using the Neighbor-Joining and the Maximum likelihood methods (Bootstrap = 1,000 replicates; other parameters: default) (Kumar et al., 2018). The phylogenetic tree we obtained was adjusted using Evolview (https://www.evolgenius.info/evolview).

### Analysis of the promoter *cis*-regulating elements

A DNA sequence 2,000 bp upstream from 12 *SmTLP* family members was extracted from the genome database and defined as the promoter sequence. This submitted to PlantCARE (http://bioinformatics.psb.ugent.be/webtools/plantcare/html/) to identify the possible cis-regulating elements in the promoter sequence (Lescot, 2002). The visualization tool TBtools was used to map the distribution of *cis*-regulating elements in the promoters (Chen et al., 2020).

### Analysis of gene expression

Two-year full-flowering *S. miltiorrhiza* plants with good and consistent growth condition were selected and divided into three groups. The control group was not given any treatment. The treatment group 1 was sprayed with 100 *μ*mol L-1 ABA and the treatment group 2 was cultured at 4 °C. Each treatment consisted of three separate biological replicates. After treatment for 6 h, samples were taken, frozen in liquid nitrogen and stored in a refrigerator at −80 °C. Total RNA was extracted from roots, stems, leaves, and flowers using RNA extraction kit (Version 1.5) (DNase I) (Biofit Biotechnologies Co., Ltd) and three biological replicates were performed. cDNA was obtained by reverse transcription using the PrimeScript™ RT Reagent Kit with gDNA Eraser (Perfect Real Time) (Takara, Beijing, China). qRT-PCR analysis was performed using SYBR Premix Ex Taq™II (TaKaRa, Japan) and the following cycling parameters: 95 °C for 30 s, and 40 cycles of 95 °C for 5 s, and 60 °C for 34 s. qRT-PCR data was calculated and analyzed using the 2 − ΔΔCt method. DNA and CDS sequences were used to design gene-specific primers for amplification (Supplementary Materials Data 2) using Primer 5 software. The error bars represent qRT-PCR result variability from the three replicates. Using the Student’s t test, *P* ≤ 0.01 (*) and *P* ≤ 0.05 (**) were compared with WT.

## Results

### Identification of SmTLPs

Based on TLP sequences known to be found in *A. thaliana* we used the online iTAK online tool to identify 12 SmTLPs in the *S. miltiorrhiza* genome. Like with previous studies on *A. thaliana*, *S. lycopersicum*, and *O. sativa*, we named the 12 *S. miltiorrhiza* TLPs (Table 1).

**Table 1:** Basic physicochemical properties of TLPs in S. miltiorrhiza.

Group	Name	Gene ID	ORF (bp)	Protein (aa)	Subcellular localization	GRAVY	Instability index		pI	Mw (kDa)
A1	TLP1	SMil_00010911	1,227	408	nucl	−0.285	58.61	unstable	9.56	45.63
	TLP2	SMil_00011940	1,050	349	chlo	−0.340	55.91	unstable	9.35	39.19
	TLP3	SMil_00005797	1,323	440	chlo	−0.211	44.69	unstable	9.52	48.65
	TLP4	SMil_00008084	1,152	383	mito	−0.588	47.96	unstable	9.35	42.57
	TLP5	SMil_00025399	1,623	540	chlo	−0.360	49.22	unstable	9.43	60.64
A2	TLP6	SMil_00009426	1,191	396	nucl	−0.317	58.59	unstable	9.35	44.02
	TLP7	SMil_00004338	1,278	425	nucl	−0.353	63.13	unstable	9.54	47.64
	TLP8	SMil_00012920	1,293	430	nucl	−0.401	65.55	unstable	9.62	48.31
B	TLP9	SMil_00019666	927	308	plas	−0.198	56.59	unstable	8.61	34.08
	TLP10	SMil_00023352	1,188	395	cyto	−0.159	53.93	unstable	8.8	43.88
	TLP11	SMil_00021823	984	327	nucl	−0.443	71.3	unstable	9.76	36.93
C	TLP12	SMil_00020329	1,227	408	nucl	−0.547	45.66	unstable	9.52	45.78

The number of amino acids in the 12 SmTLPs ranged from 308 to 540. Physicochemical property analysis results revealed that the relative molecular weight of the 12 TLPs ranged from 34.08 to 60.64 kDa. The theoretical isoelectric points (*p* I) of all 12 TLPs were greater than 7, with TLP9 having the smallest *p* I (8.61) and TLP11 having the largest pI (9.76). The instability indice values for all *SmTLPs* were greater than 40, indicating that they are unstable proteins. In the grand average of hydropathicity, all SmTLPs’ values were negative. This indicates that SmTLPs are hydrophilic proteins. TLP4 had the best water solubility with a GRAVY value of -0.588. Subcellular localization prediction showed that all SmTLPs were located in the nucleus, suggesting that they play an important role there. This is consistent with previous studies on *A. thaliana* and *S. lycopersicum* (Lai et al., 2004; Zhang et al., 2020). The basic physicochemical properties of TLPs are shown in Table 1.

### Phylogenetic analysis of SmTLPs

**Figure 1:**
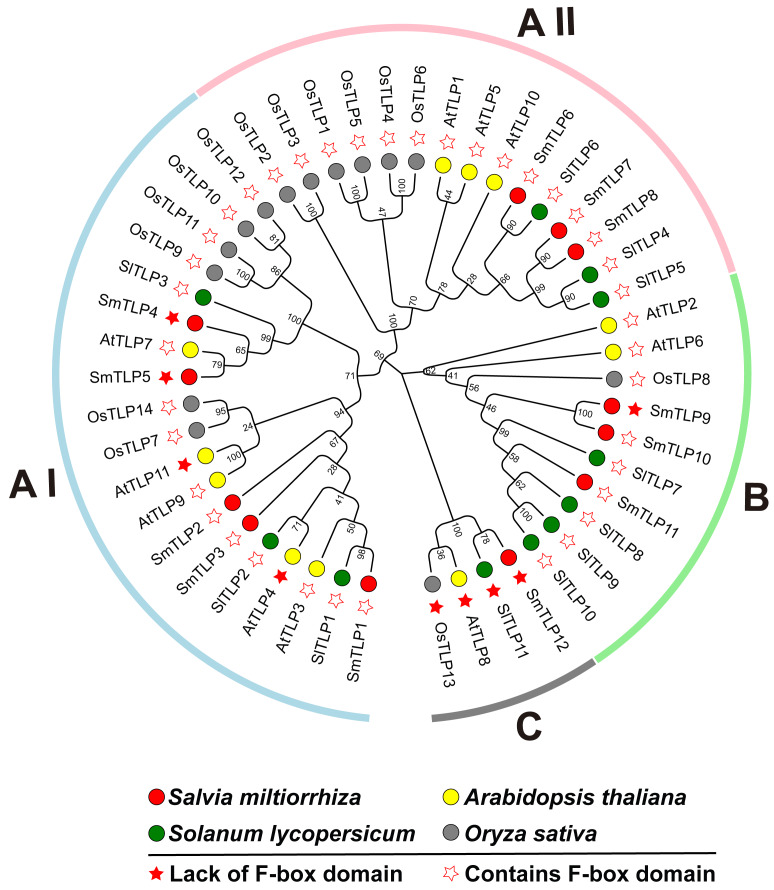
The phylogenetic tree of TLP proteins in four plants, including *A. thaliana*, *O. sativa*, *S. lycopersicum*, and *S. miltiorrhiza*. The phylogenetic tree is constructed by MEGA X with the following parameters: Neighbor-Joining method, Poisson correction, Bootstrap = 1,000 replicates. All the members were divided into four classes which were represented by different colors. The members from various species were indicated by different labels. The proteins with F-box and those without F-box were marked by different stars.

To investigate the phylogenetic relationship between *S. miltiorrhiza* TLPs, we constructed a phylogenetic tree including *S. miltiorrhiza*, *A. thaliana*, *S. lycopersicum*, and *O. sativa* TLPs using the Neighbor-Joining and Maximum likelihood methods (Fig. 1). This analysis clearly reveals that the 12 SmTLPs, 11 AtTLPs, 11 SlTLPs, and 14 OsTLPs are clustered and divided into three large subfamilies: A, B, and C. The A subfamily is further divided into AI and AII groups. This grouping is consistent with previous reports on *S. lycopersicum* (Zhang et al., 2020). The AI subfamily contains the most TLPs, including five SmTLPs (SmTLP1, SmTLP2, SmTLP3, SmTLP4, and SmTLP5) and 14 other TLPs. There are 15 TLPs in the A II subfamily, including three SmTLPs (SmTLP6, SmTLP7, and SmTLP8). The B subfamily has three SmTLPs (SmTLP9, SmTLP10, and SmTLP11). The C subfamily is the smallest subfamily, with only four TLPs (OsTLP13, AtTLP8, SlTLP11, and SmTLP12), and none of these TLPs contain F-box domains. The TLPs within each subfamily have high homology and close evolutionary relationships (Wang, Xu & Kong, 2018). However, the evolutionary concerns of *S. miltiorrhiza*, *A. thaliana*, and *S. lycopersicum* are closer.

**Figure 2:**
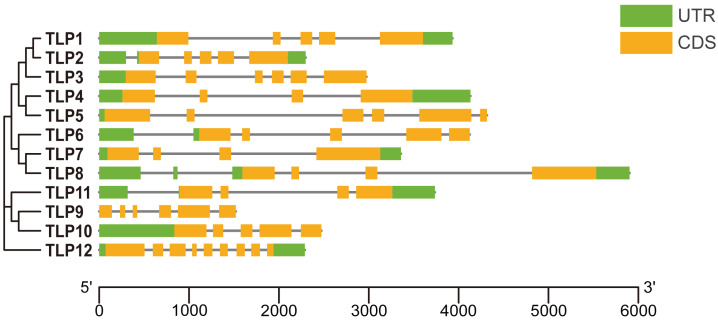
The gene structure of *TLP genes* in *S. miltiorrhiza*. UTR, Untranslated region; CDS, Coding sequence. The picture is drawn by Tbtools.

### Gene structure, conserved motifs, and conserved domains in SmTLPs

To improve our understanding of gene function and evolution, we analyzed the *TLP* gene structure (Fig. 2). Although some SmTLPs are very close phylogenetically, their exon number, distribution, and gene length greatly differ, especially between *TLP1* and *TLP2*, and between *TLP7* and *TLP8*. This is consistent with previous studies on *S. lycopersicum*, and may imply that the genetic structure of *TLPs* is easily altered (Zhang et al., 2020).

**Figure 3:**
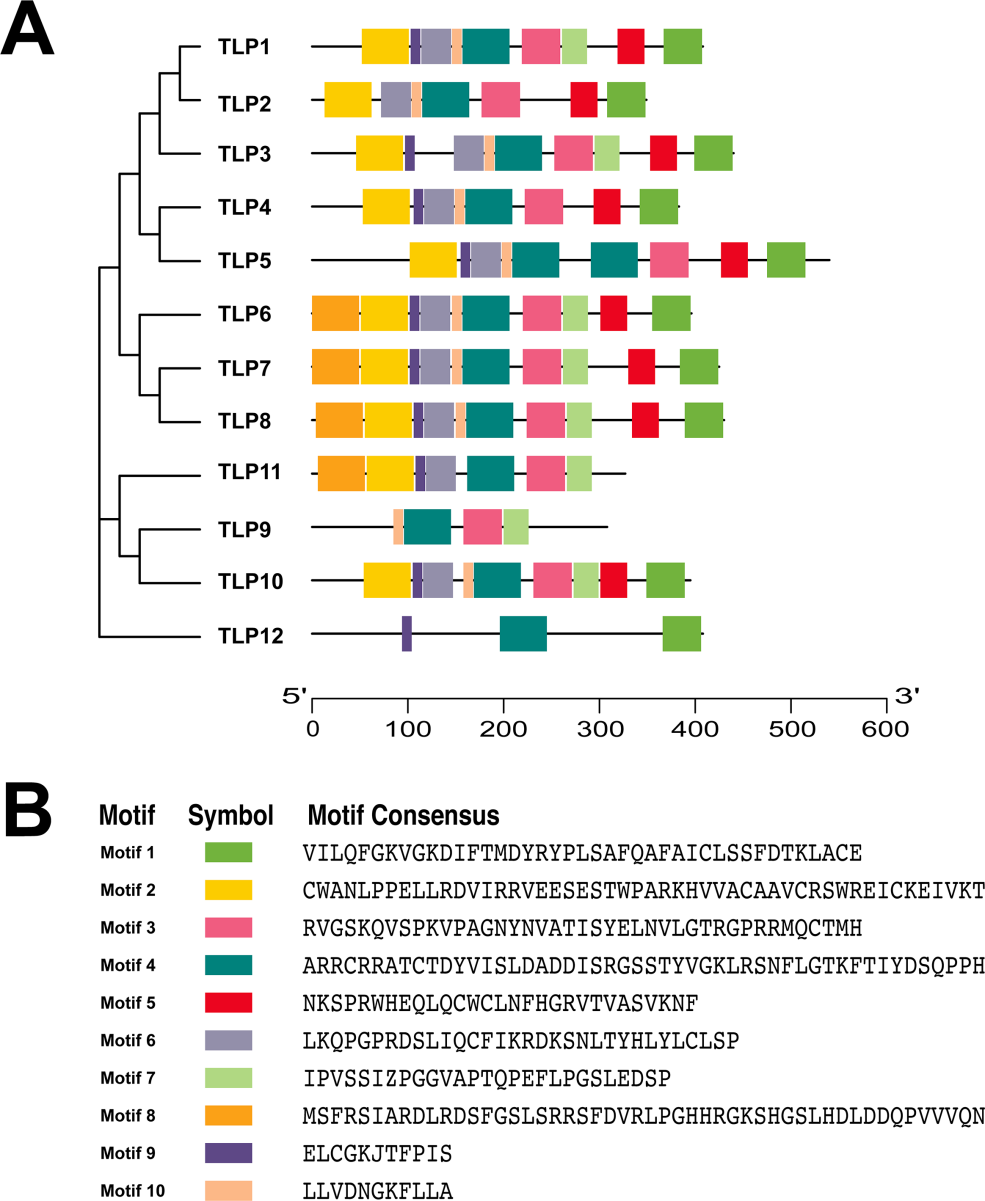
The conserved motifs of TLP proteins in *S. miltiorrhiza*. (A) The localization of conserved motifs; (B) the Motif consensuses of conserved motifs. The full-length protein of SmTLPs was analyzed by MEME online software (maximum number of 10 motifs; minimum motif width of 6; maximum motif width of 200).

To further understand the process of TLP evolution, we analyzed the TLP motifs using MEME. A total of 10 motifs were identified (Fig. 3 and Fig. S1).

Conservative motif analysis results showed that all SmTLP members contained motif 1 and motif 4 (Fig. 3A). SmTLP members on the same branch of the phylogenetic tree had small differences in motif type, number, and position. SmTLP members on different branches of the phylogenetic tree had larger differences in motif type, number, and position. In particular, TLP12 had the fewest motifs, which may indicate that it has other unique functions. Closely related members on the phylogenetic tree often contain the same motif, indicating that TLPs in the same group have similar functions.

**Figure 4:**
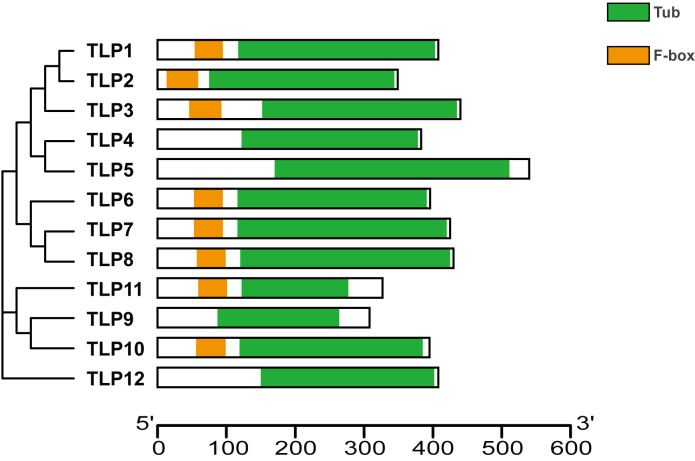
The conserved domains of TLP proteins in *S. miltiorrhiza*. Different domains, including Tub and F-box were represented by colorful blocks. The relevant analysis results are based on the Pfam database.

Proteins’ key conserved domains often confer function. To explore SmTLP function and structure we used the SMART database. The results show that, except for SmTLP9 and SmTLP12, SmTLPs have both a Tub domain (PF01167) and an F-box domain (PF00646) (Fig. 4 and Fig. S2). SmTLP structure was further explored by Tub domain 3D modeling. This analysis revealed that SmTLP11 Tub structure is incomplete, which may impact its function.

**Figure 5:**
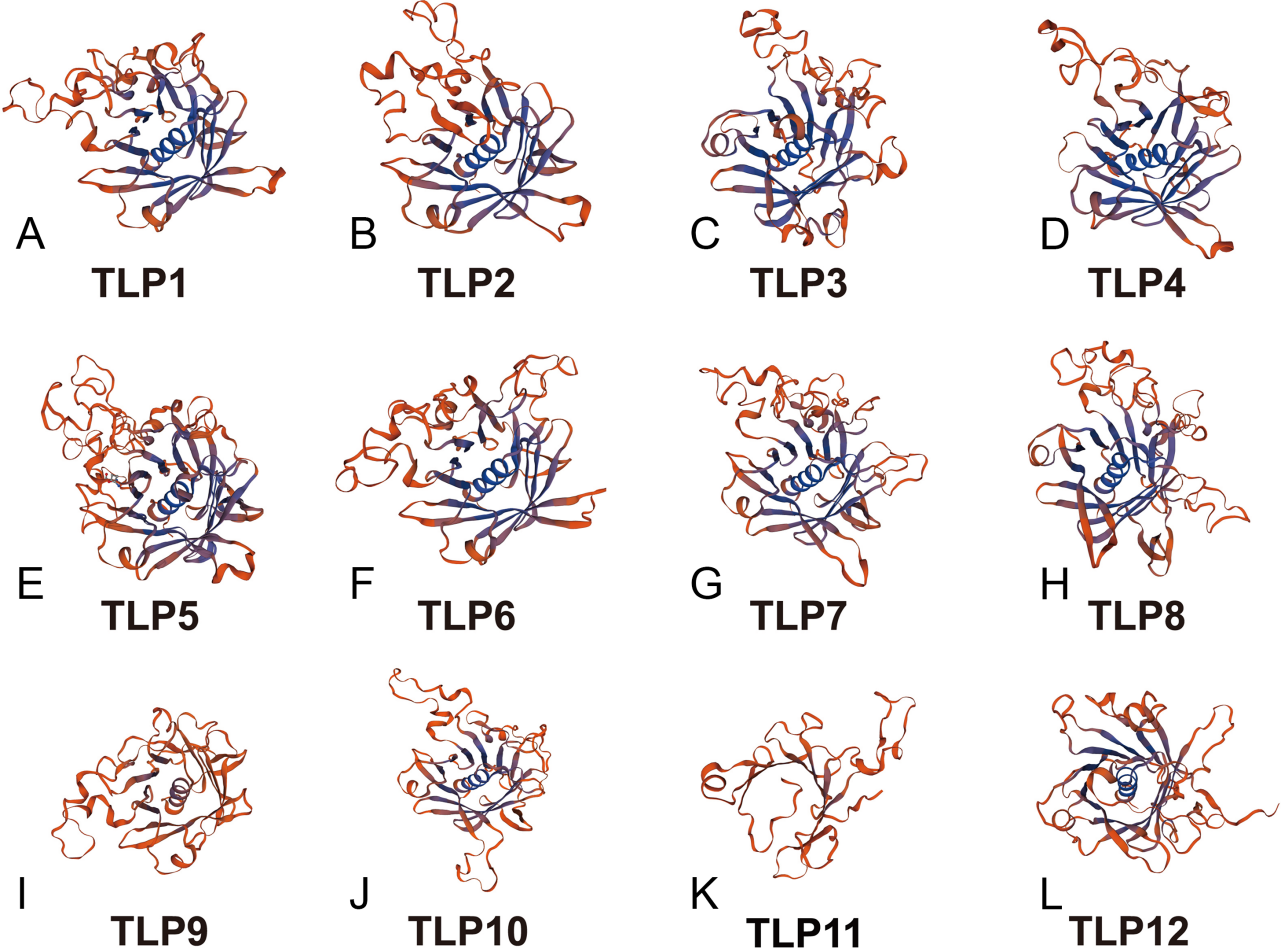
The 3D-model of the Tub domain of TLP proteins in *S. miltiorrhiza*. The analysis results and pictures are generated by SWISS-MODEL (default parameters).

With additional 3D modeling and analysis of the TLPs’ Tub domain, we found that 11 SmTLPs have a complete Tub domain consisting of a closed *β* barrel with 12 anti-parallel strands and a central hydrophobic *α* helix (Fig. 5). We also found that TLP11 has an incomplete Tub domain, which may account for its special function, but this requires further experimental verification.

### Analysis of SmTLP promoter sequences

**Figure 6:**
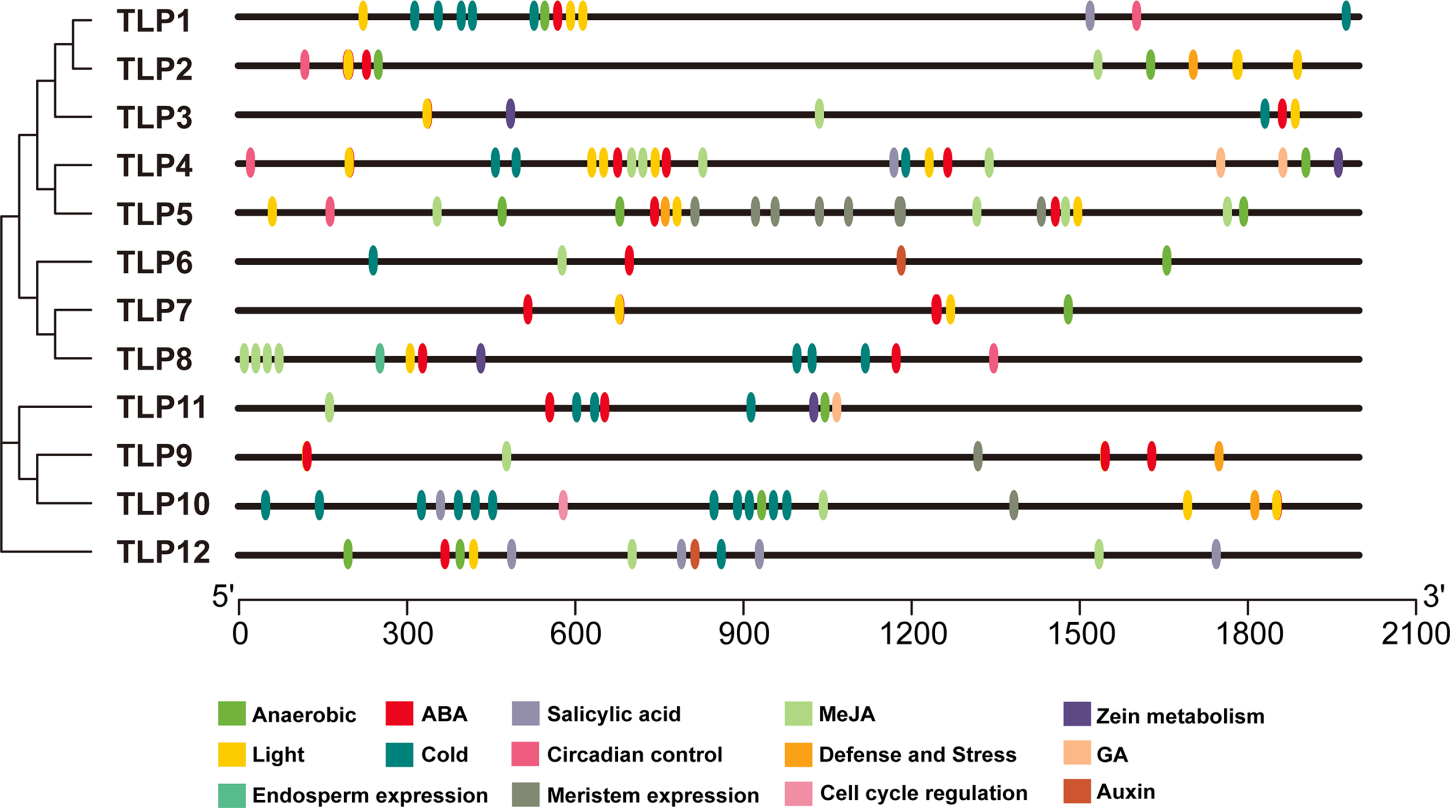
The *cis*-element distribution in the putative promoters of *TLP* genes in *S. miltiorrhiza*. These *cis*-elements were shown in different colors and were located at 2,000 bp upstream of the *SmTLP* promoters.

**Figure 7:**
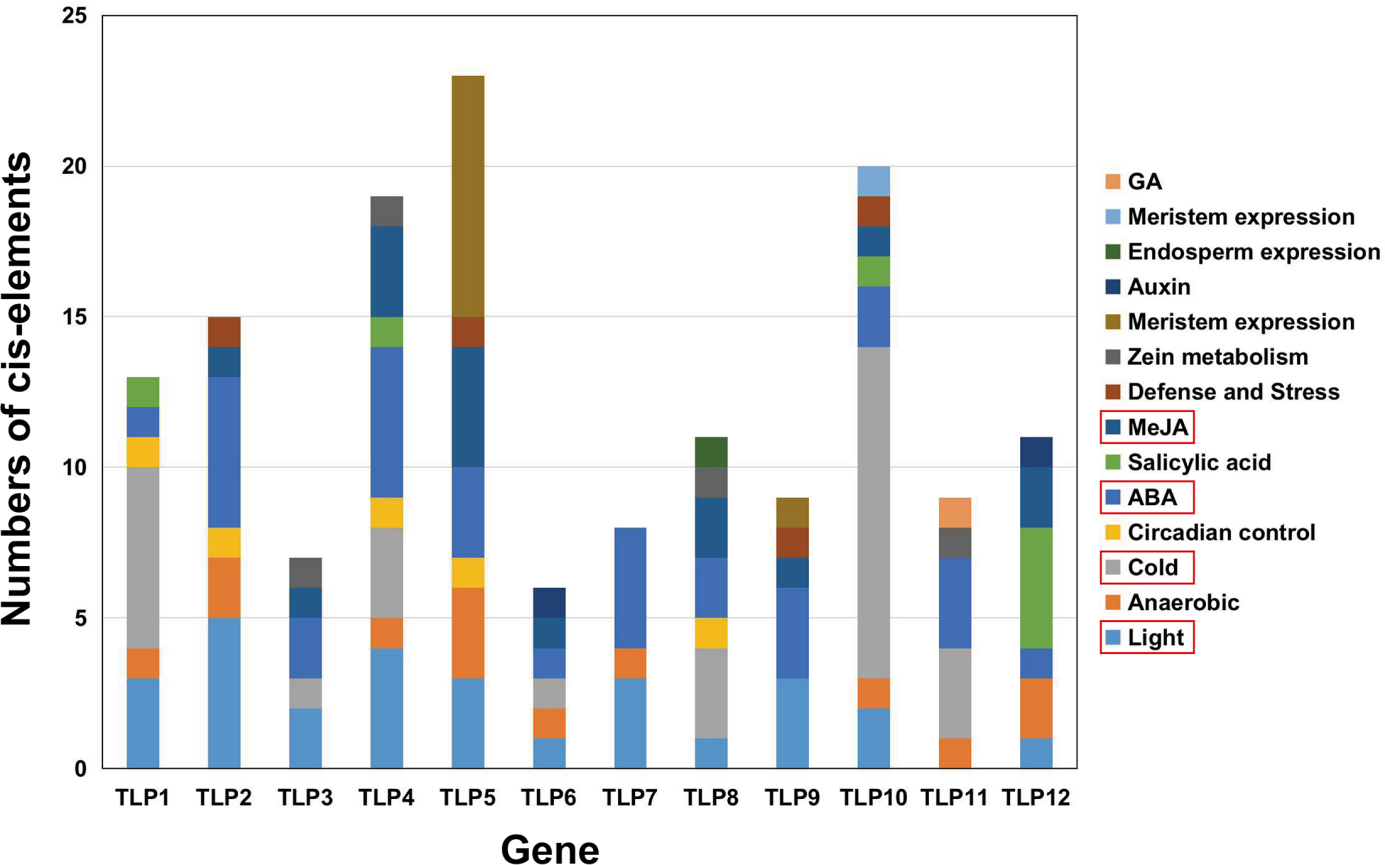
The analysis of putative *Cis*-elements of *TLP* genes in *S. miltiorrhiza*. These *cis*-elements were shown in different colors.

The PlantCARE database was used to analyze 2,000 bp of DNA sequence upstream of the *TLP* genes’ transcription start site to determine the potential functions of TLPs. The main promoter was mapped and is displayed in Fig. 6. A variety of cis-elements that respond to environmental and hormonal signals have been identified in SmTLPs (Table 2). This implies that SmTLPs have a somewhat complicated mechanism of expression regulation. TLPs contain a variety of hormone response elements, including ABA, auxin, gibberellin, and MeJA response elements. Previous studies have shown that TLPs can also respond to light, temperature, hypoxia, and salt stress (Zhang et al., 2020). SmTLP10 had the most low-temperature response elements, which may indicate that it plays an important role in plant cold resistance (Fig. 7). TLP5 had the most meristem expression elements, indicating that it is important for plant meristem growth. Additional experiments are required to confirm these inferences.

### The expression profiling of SmTLPs in different tissues of *S. miltiorrhiza*

**Figure 8:**
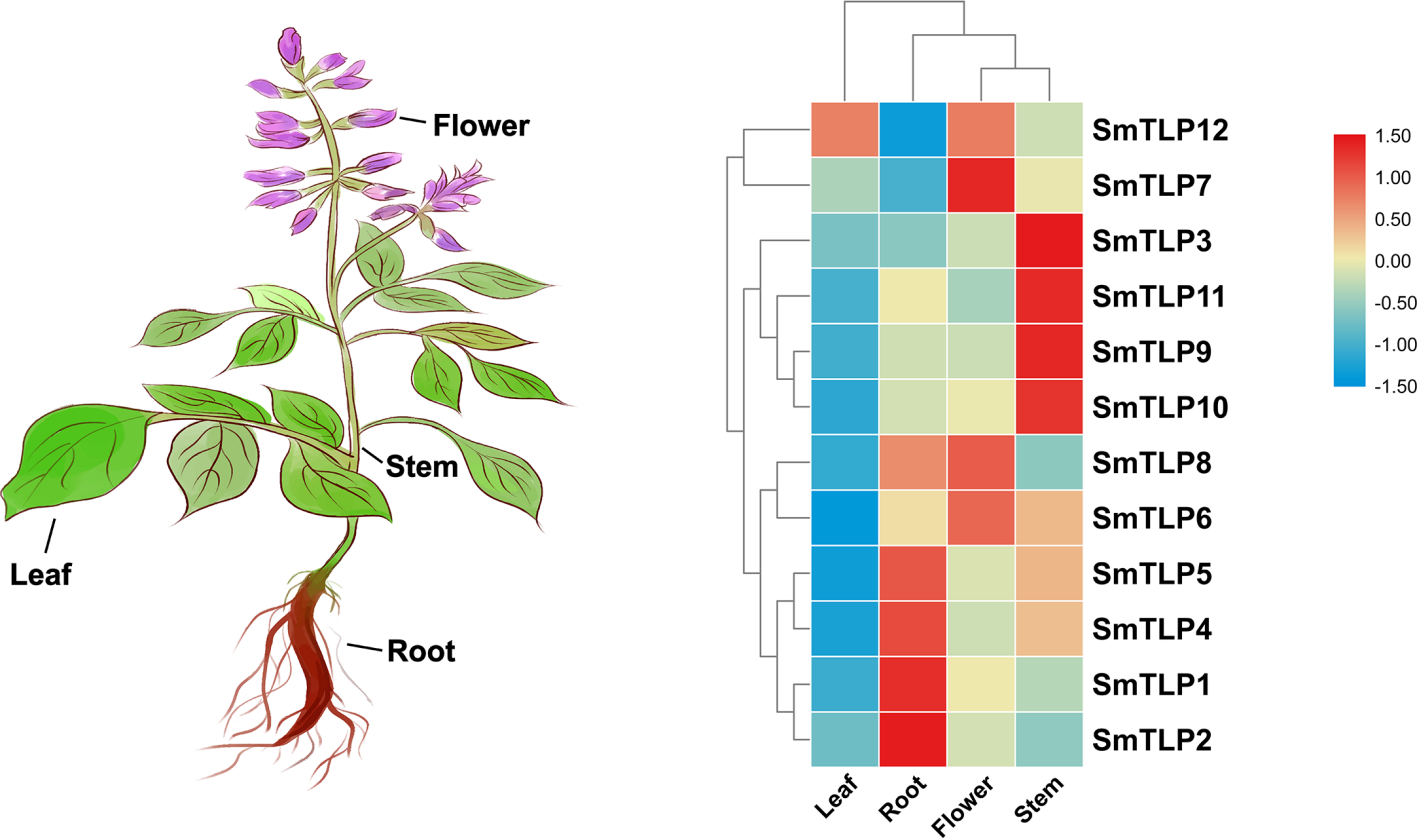
The expression patterns of *TLP* genes in different *S. miltiorrhiza* tissues, including leaf, root, flower, and stem. The data was homogenized by Log_2_ (*X* + 1) and the heat map was drawn by TBtools.

Tissue-specific gene expression can be used to predict gene function. Therefore, we used qRT-PCR to detect *SmTLP* expression in various *S. miltiorrhiza* tissues (Fig. 8). The results showed that *SmTLPs* have distinct expression patterns. Four *SmTLPs* (*SmTLP1*, *SmTLP2*, *SmTLP4*, and *SmTLP5*) have unique expression patterns in the roots, which may imply that they function in stress resistance. This speculation requires further experimental verification. Four *SmTLPs* (*SmTLP3*, *SmTLP9*, *SmTLP10*, and *SmTLP11*) have unique expression patterns in the stems. The remaining four *SmTLPs* (*SmTLP6*, *SmTLP7*, *SmTLP8*, and *SmTLP12*) have unique expression patterns mainly in the flowers. Only *SmTLP12* is expressed in leaves.

**Table 2:** The informations of *cis* elements.

Gene	*Cis*-elements	Sequences	Correlation
TLP1	ARE	AAACCA	Anaerobic Induction
TLP1	ACE	GACACGTATG	Light
TLP1	G-Box	CACGTT	Light
TLP1	G-box	CACGAC	Light
TLP1	circadian	CAAAGATATC	Circadian Control
TLP1	LTR	CCGAAA	Low-Temperature
TLP1	LTR	CCGAAA	Low-Temperature
TLP1	LTR	CCGAAA	Low-Temperature
TLP1	LTR	CCGAAA	Low-Temperature
TLP1	LTR	CCGAAA	Low-Temperature
TLP1	LTR	CCGAAA	Low-Temperature
TLP1	ABRE	ACGTG	Abscisic Acid
TLP1	TCA-element	CCATCTTTTT	Salicylic Acid
TLP2	ABRE	GACACGTGGC	Abscisic Acid
TLP2	ABRE	CACGTG	Abscisic Acid
TLP2	ABRE	ACGTG	Abscisic Acid
TLP2	ABRE	AACCCGG	Abscisic Acid
TLP2	ABRE	ACGTG	Abscisic Acid
TLP2	G-box	ACACGTGGC	Light
TLP2	G-box	CACGTG	Light
TLP2	G-box	GCCACGTGGA	Light
TLP2	G-box	CACGAC	Light
TLP2	CGTCA-motif	CGTCA	Meja
TLP2	circadian	CAAAGATATC	Circadian Control
TLP2	TC-rich repeats	ATTCTCTAAC	Defense And Stress
TLP2	TGACG-motif	TGACG	Meja
TLP2	ARE	AAACCA	Anaerobic Induction
TLP2	ARE	AAACCA	Anaerobic Induction
TLP2	G-Box	CACGTG	Light
TLP2	G-Box	CACGTT	Light
TLP3	TGACG-motif	TGACG	Meja
TLP3	O2-site	GATGACATGG	Zein Metabolism Regulation
TLP3	ABRE	ACGTG	Abscisic Acid
TLP3	ABRE	ACGTG	Abscisic Acid
TLP3	CGTCA-motif	CGTCA	Meja
TLP3	LTR	CCGAAA	Low-Temperature
TLP3	G-box	TACGTG	Light
TLP3	G-box	TACGTG	Light
TLP4	TGACG-motif	TGACG	Meja
TLP4	TGACG-motif	TGACG	Meja
TLP4	TGACG-motif	TGACG	Meja
TLP4	ARE	AAACCA	Anaerobic Induction
TLP4	G-Box	CACGTG	Light
TLP4	TATC-box	TATCCCA	Gibberellin
TLP4	TATC-box	TATCCCA	Gibberellin
TLP4	TCA-element	TCAGAAGAGG	Salicylic Acid
TLP4	O2-site	GATGACATGG	Zein Metabolism Regulation
TLP4	ABRE	CACGTG	Abscisic Acid
TLP4	ABRE	ACGTG	Abscisic Acid
TLP4	ABRE	ACGTG	Abscisic Acid
TLP4	ABRE	ACGTG	Abscisic Acid
TLP4	ABRE	ACGTG	Abscisic Acid
TLP4	G-box	CACGTG	Light
TLP4	G-box	CCACGTAA	Light
TLP4	G-box	TACGTG	Light
TLP4	G-box	CACGTC	Light
TLP4	G-box	CACGTC	Light
TLP4	CGTCA-motif	CGTCA	Meja
TLP4	CGTCA-motif	CGTCA	Meja
TLP4	CGTCA-motif	CGTCA	Meja
TLP4	LTR	CCGAAA	Low-Temperature
TLP4	LTR	CCGAAA	Low-Temperature
TLP4	LTR	CCGAAA	Low-Temperature
TLP4	circadian	CAAAGATATC	Circadian Control
TLP5	TC-rich repeats	ATTCTCTAAC	Defense And Stress
TLP5	TGACG-motif	TGACG	Meja
TLP5	TGACG-motif	TGACG	Meja
TLP5	TGACG-motif	TGACG	Meja
TLP5	TGACG-motif	TGACG	Meja
TLP5	ARE	AAACCA	Anaerobic Induction
TLP5	ARE	AAACCA	Anaerobic Induction
TLP5	ARE	AAACCA	Anaerobic Induction
TLP5	CAT-box	GCCACT	Meristem Expression
TLP5	CAT-box	GCCACT	Meristem Expression
TLP5	CAT-box	GCCACT	Meristem Expression
TLP5	CAT-box	GCCACT	Meristem Expression
TLP5	CAT-box	GCCACT	Meristem Expression
TLP5	CAT-box	GCCACT	Meristem Expression
TLP5	CAT-box	GCCACT	Meristem Expression
TLP5	CAT-box	GCCACT	Meristem Expression
TLP5	G-Box	CACGTT	Light
TLP5	ABRE	ACGTG	Abscisic Acid
TLP5	ABRE	ACGTG	Abscisic Acid
TLP5	ABRE	ACGTG	Abscisic Acid
TLP5	G-box	CACGTC	Light
TLP5	G-box	CACGTC	Light
TLP5	circadian	CAAAGATATC	Circadian Control
TLP5	CGTCA-motif	CGTCA	Meja
TLP5	CGTCA-motif	CGTCA	Meja
TLP5	CGTCA-motif	CGTCA	Meja
TLP5	CGTCA-motif	CGTCA	Meja
TLP6	AuxRR-core	GGTCCAT	Auxin
TLP6	TGACG-motif	TGACG	Meja
TLP6	ARE	AAACCA	Anaerobic Induction
TLP6	G-Box	CACGTT	Light
TLP6	ABRE	ACGTG	Abscisic Acid
TLP6	CGTCA-motif	CGTCA	Meja
TLP6	LTR	CCGAAA	Low-Temperature
TLP7	G-Box	CACGTT	Light
TLP7	ARE	AAACCA	Anaerobic Induction
TLP7	ABRE	ACGTG	Abscisic Acid
TLP7	ABRE	ACGTG	Abscisic Acid
TLP7	ABRE	CGTACGTGCA	Abscisic Acid
TLP7	ABRE	ACGTG	Abscisic Acid
TLP7	G-box	CACGTC	Light
TLP7	G-box	TACGTG	Light
TLP8	CGTCA-motif	CGTCA	Meja
TLP8	CGTCA-motif	CGTCA	Meja
TLP8	circadian	CAAAGATATC	Circadian Control
TLP8	LTR	CCGAAA	Low-Temperature
TLP8	LTR	CCGAAA	Low-Temperature
TLP8	LTR	CCGAAA	Low-Temperature
TLP8	G-box	TACGTG	Light
TLP8	O2-site	GATGA(C/T)(A/G)TG(A/G)	Zein Metabolism Regulation
TLP8	ABRE	ACGTG	Abscisic Acid
TLP8	ABRE	GACACGTGGC	Abscisic Acid
TLP8	ACE	GACACGTATG	Light
TLP8	GCN4_motif	TGAGTCA	Endosperm Expression
TLP8	TGACG-motif	TGACG	Meja
TLP8	TGACG-motif	TGACG	Meja
TLP9	CAT-box	GCCACT	Meristem Expression
TLP9	G-Box	CACGTT	Light
TLP9	G-Box	CACGTT	Light
TLP9	TC-rich repeats	ATTCTCTAAC	Defense And Stress
TLP9	TGACG-motif	TGACG	Meja
TLP9	G-box	TACGTG	Light
TLP9	G-box	TAAACGTG	Light
TLP9	CGTCA-motif	CGTCA	Meja
TLP9	ABRE	ACGTG	Abscisic Acid
TLP9	ABRE	ACGTG	Abscisic Acid
TLP9	ABRE	ACGTG	Abscisic Acid
TLP10	TC-rich repeats	ATTCTCTAAC	Defense And Stress
TLP10	MSA-like	TCCAACGGT	Cell Cycle Regulation
TLP10	TGACG-motif	TGACG	Meja
TLP10	TCA-element	CCATCTTTTT	Salicylic Acid
TLP10	ABRE	ACGTG	Abscisic Acid
TLP10	ABRE	ACGTG	Abscisic Acid
TLP10	ARE	AAACCA	Anaerobic Induction
TLP10	CAT-box	GCCACT	Meristem Expression
TLP10	G-Box	CACGTT	Light
TLP10	G-box	TAAACGTG	Light
TLP10	G-box	TACGTG	Light
TLP10	CGTCA-motif	CGTCA	Meja
TLP10	LTR	CCGAAA	Low-Temperature
TLP10	LTR	CCGAAA	Low-Temperature
TLP10	LTR	CCGAAA	Low-Temperature
TLP10	LTR	CCGAAA	Low-Temperature
TLP10	LTR	CCGAAA	Low-Temperature
TLP10	LTR	CCGAAA	Low-Temperature
TLP10	LTR	CCGAAA	Low-Temperature
TLP10	LTR	CCGAAA	Low-Temperature
TLP10	LTR	CCGAAA	Low-Temperature
TLP10	LTR	CCGAAA	Low-Temperature
TLP10	LTR	CCGAAA	Low-Temperature
TLP11	TATC-box	TATCCCA	Gibberellin
TLP11	ARE	AAACCA	Anaerobic Induction
TLP11	TGACG-motif	TGACG	Meja
TLP11	CGTCA-motif	CGTCA	Meja
TLP11	LTR	CCGAAA	Low-Temperature
TLP11	LTR	CCGAAA	Low-Temperature
TLP11	LTR	CCGAAA	Low-Temperature
TLP11	O2-site	GATGATGTGG	Zein Metabolism Regulation
TLP11	ABRE	AACCCGG	Abscisic Acid
TLP11	ABRE	GCAACGTGTC	Abscisic Acid
TLP11	ABRE	AACCCGG	Abscisic Acid
TLP12	ARE	AAACCA	Anaerobic Induction
TLP12	ARE	AAACCA	Anaerobic Induction
TLP12	AuxRR-core	GGTCCAT	Auxin
TLP12	TGACG-motif	TGACG	Meja
TLP12	TGACG-motif	TGACG	Meja
TLP12	G-box	CACGTC	Light
TLP12	CGTCA-motif	CGTCA	Meja
TLP12	CGTCA-motif	CGTCA	Meja
TLP12	LTR	CCGAAA	Low-Temperature
TLP12	TCA-element	CCATCTTTTT	Salicylic Acid
TLP12	TCA-element	CCATCTTTTT	Salicylic Acid
TLP12	TCA-element	CCATCTTTTT	Salicylic Acid
TLP12	TCA-element	CCATCTTTTT	Salicylic Acid
TLP12	ABRE	ACGTG	Abscisic Acid

### The expression of SmTLPs under ABA and cold treatment

**Figure 9:**
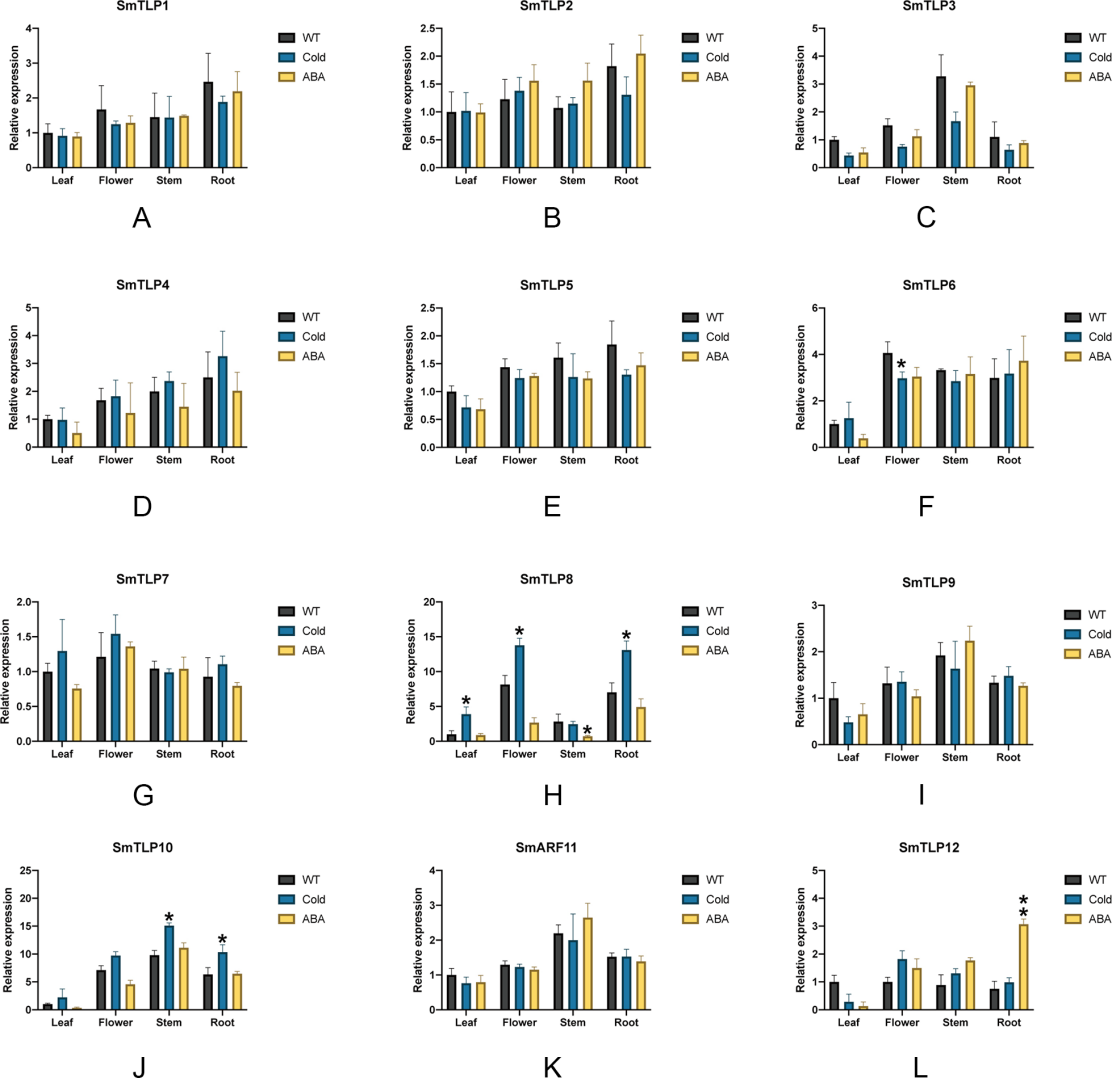
The expression of *TLP* genes in *S. miltiorrhiza* under cold stress and exogenous ABA stress treatment (A-L). WT, Wild type; Cold, Exposure at 4 °C for 6 h; ABA, Exposure to 100 µM ABA for 6 h. Error bars represent the variability of qRT-PCR results from three replicates. **, *P* ≤ 0.01 and *, *P* ≤ 0.05, based on Student’s *t* test, compared with WT.

We performed stress treatment on *S. miltiorrhiza* and used qRT-PCR to detect *SmTLP* expression to explore the response of *SmTLPs* to stress (Fig. 9). Under cold stress, the stem *SmTLP3* expression decreased significantly, while *SmTLP10* stem expression increased significantly. This suggests that these genes may play important roles in stem growth. After the flowers were subjected to cold stress, *SmTLP6* expression decreased significantly and *SmTLP8* expression increased significantly. This may indicate that they are involved in important physiological processes in flowers. Under cold stress, the *SmTLP8* and *SmTLP10* expression in roots significantly increased, indicating that they are involved in the cold-stress response in *S. miltiorrhiza*. Under ABA stress, *SmTLP12* expression significantly increased, indicating that *SmTLP12* may participate in the ABA pathway in *S. miltiorrhiza*.

## Discussion

TLP, a transcription factor widely found in animal and plant genomes, plays a variety of important functions (Wang, Xu & Kong, 2018). To date, analysis of plant *TLP* gene families has shown that multiple TLP genes are involved in plant responses to biological and abiotic stresses (Lai et al., 2004; Liu, 2008; Xu et al., 2016c; Yulong et al., 2016). This indicates that *TLP* genes can be used as candidate genes in plant resistance breeding. *S. miltiorrhiza* is an important herbal medicine and is widely cultivated because of its high economic value and importance in research. In this study, we examined the potential functions of *S. miltiorrhiza* TLPs. Using bioinformatics, we identified 12 *SmTLPs* and conducted comprehensive analyses including gene structure, protein structure, phylogenetic, and expression analyses.

Analysis of gene and protein structure revealed that most TLPs have both a Tub and F-box domain and is consistent with previously reported results (Lai et al., 2004; Liu, 2008; Xu et al., 2016c; Yulong et al., 2016). Similarly, based on the 3D modeling approach, we confirm that SmTLPs, except for SMTLP11, have the complete TUB domain consisting of a closed *β* barrel with 12 anti-parallel strands and a central hydrophobic *α* helix. This suggests that SMTLP11 may have special qualities.

Phylogenetic analysis showed that the 12 identified SmTLPs are divided into three subfamilies (Fig. 5). This is consistent with the results of previous studies, indicating the conservation and importance of TLPs (Xu et al., 2016b; Yulong et al., 2016; Zhang et al., 2020). Interestingly, we found that none of the TLPs in the C subfamily possess the F-box domain thought to be related to plant stress resistance (Gagne et al., 2002; Lai et al., 2004; Xu et al., 2019). This result indicates that TLPs in the C subfamily are highly conserved, and suggests that they may have other important functions in plants.

The cis-element is a regulatory element in plants that plays a vital role in transcription level regulation. Promoter analysis suggests that *SmTLPs* not only participate in hormone regulation, but may also play an important role in plant resistance to drought, low temperature, and exogenous ABA responses (Fig. 6 and Fig. 7) (Bao et al., 2014). We found that SmTLP10 has the most cold-induced cis-elements, implying that it plays an important role in the response to cold in *S. miltiorrhiza*.

To further study the response of *SmTLPs* to abiotic stress, we used q-PCR to analyze the expression patterns of 12 *SmTLPs* in various *S. miltiorrhiza* tissues. The results showed that *SmTLPs* are expressed in different tissues. Additionally, we found that, consistent with our promoter analysis, the *SmTLP10* expression increased significantly in roots and stems after *S. miltiorrhiza* was exposed to cold stress. This indicates that *SmTLP10* plays an important role in plant resistance to cold stress. Moreover, *SmTLP12* expression increased significantly in roots after being induced by exogenous ABA. This may indicate that *SmTLP12* is involved in the ABA pathway in *S. miltiorrhiza*.

## Conclusions

In this paper, we identified 12 TLPs in *S. miltiorrhiza* and conducted a comprehensive analysis of SmTLPs, including gene structure analysis, protein structure analysis, phylogenetic analysis and expression analysis, etc. Promoter analysis shows that most SmTLPs are not only related to hormone response, but also related to abiotic stress. Expression analysis showed that the 12 SmTLPs were mainly divided into three categories: one was specifically expressed in roots, one was specifically expressed in stems, and the other was specifically expressed in leaves. Additional studies have shown that SmTLP10 may play an important role in plants’ resistance to cold, while *SmTLP12* may be involved in the plant’s ABA metabolic pathway. Our results clarify the basic bioinformatic characteristics of TLPs in *S. miltiorrhiza* and verify that some TLPs (*TLP10* and *TLP12*) play an important role in the anti-stress functions of *S. miltiorrhiza*. This work also lays the foundation for stress resistance breeding in *S. miltiorrhiza*.

##  Supplemental Information

10.7717/peerj.11403/supp-1 Supplemental Information 1 SmTLPs Motif LOGO.

10.7717/peerj.11403/supp-2 Supplemental Information 2 Amino acid sequence alignment of SmTLPs.

10.7717/peerj.11403/supp-3 Supplemental Information 3 TLPs in other plants.

10.7717/peerj.11403/supp-4 Supplemental Information 4 The primers used for qPCR analysis.

10.7717/peerj.11403/supp-5 Supplemental Information 5 The raw data for 22 selected SmTLPs RT-qPCR analysis

## Additional Information and Declarations

### Data Availability

The following information was supplied regarding data availability: The genome data of *Salvia miltiorrhiza* are stored in the China National Traditional Chinese Medicine Data Center (http://www.ndctcm.org/shujukujieshao/2015-04-23/27.html) and can be downloaded via FTP software.
