# Supplementary figures and images for: Genome-wide identification of the Tubby-Like Protein (TLPs) family in medicinal model plant Salvia miltiorrhiza
Source: PeerJ. 2021 May 12;9:e11403. doi: 10.7717/peerj.11403 (PMC8123234; doi:10.7717/peerj.11403)

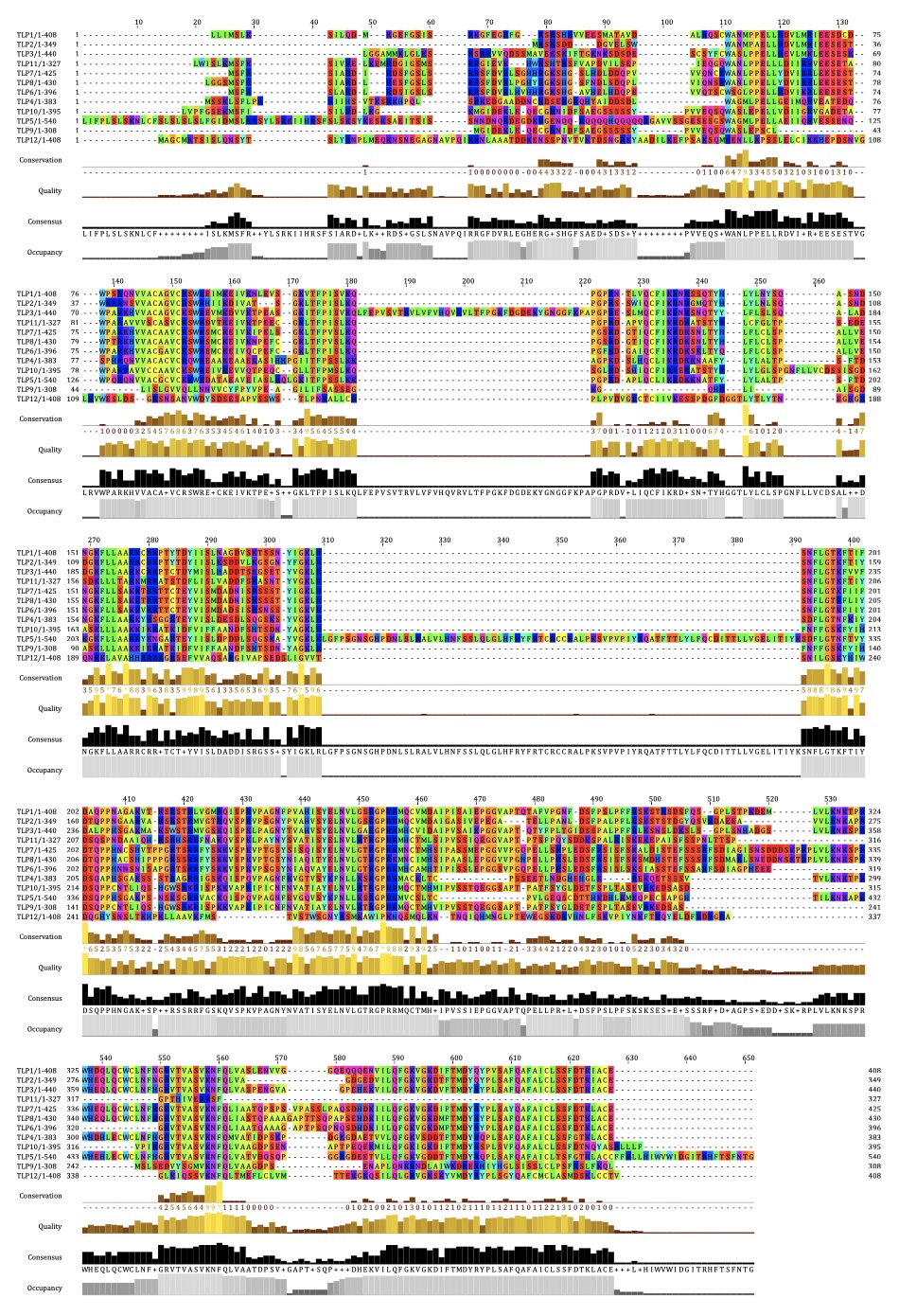

Supplement: Supplemental Information 2 [file peerj-09-11403-s002.jpg]
